# A four-step strategy for handling missing outcome data in randomised trials affected by a pandemic

**DOI:** 10.1186/s12874-020-01089-6

**Published:** 2020-08-12

**Authors:** Suzie Cro, Tim P. Morris, Brennan C. Kahan, Victoria R. Cornelius, James R. Carpenter

**Affiliations:** 1 Imperial Clinical Trials Unit, Imperial College London, Stadium House, 68 Wood Lane, London, UK; 2 Department of Medical Statistics, London School of Hygiene and Tropical Medicine, London, UK; 3 MRC Clinical Trials Unit at UCL, 90 High Holborn, London, UK

**Keywords:** Missing data, Pandemic, Coronavirus SARS-CoV-2, Covid-19, Randomised trials, Estimands, Sensitivity analysis, Controlled multiple imputation

## Abstract

**Background:**

The coronavirus pandemic (Covid-19) presents a variety of challenges for ongoing clinical trials, including an inevitably higher rate of missing outcome data, with new and non-standard reasons for missingness. International drug trial guidelines recommend trialists review plans for handling missing data in the conduct and statistical analysis, but clear recommendations are lacking.

**Methods:**

We present a four-step strategy for handling missing outcome data in the analysis of randomised trials that are ongoing during a pandemic. We consider handling missing data arising due to (i) participant infection, (ii) treatment disruptions and (iii) loss to follow-up. We consider both settings where treatment effects for a ‘pandemic-free world’ and ‘world including a pandemic’ are of interest.

**Results:**

In any trial, investigators should; (1) Clarify the treatment estimand of interest with respect to the occurrence of the pandemic; (2) Establish what data are missing for the chosen estimand; (3) Perform primary analysis under the most plausible missing data assumptions followed by; (4) Sensitivity analysis under alternative plausible assumptions. To obtain an estimate of the treatment effect in a ‘pandemic-free world’, participant data that are clinically affected by the pandemic (directly due to infection or indirectly via treatment disruptions) are not relevant and can be set to missing. For primary analysis, a missing-at-random assumption that conditions on all observed data that are expected to be associated with both the outcome and missingness may be most plausible. For the treatment effect in the ‘world including a pandemic’, all participant data is relevant and should be included in the analysis. For primary analysis, a missing-at-random assumption – potentially incorporating a pandemic time-period indicator and participant infection status – or a missing-not-at-random assumption with a poorer response may be most relevant, depending on the setting. In all scenarios, sensitivity analysis under credible missing-not-at-random assumptions should be used to evaluate the robustness of results. We highlight controlled multiple imputation as an accessible tool for conducting sensitivity analyses.

**Conclusions:**

Missing data problems will be exacerbated for trials active during the Covid-19 pandemic. This four-step strategy will facilitate clear thinking about the appropriate analysis for relevant questions of interest.

## Background

On 11th March 2020 the World Health Organisation declared the novel coronavirus (SARS-CoV-2) outbreak as a pandemic (Covid-19) [1]. Covid-19 presents a variety of challenges for the conduct and analysis of ongoing clinical trials. This has been recognised by the FDA, EMA and MHRA, who have issued guidance on trial conduct during Covid-19 [2–4]. Subject to participant and investigator safety, it is recommended that data collection continue for as long as possible, but where feasible this is to be undertaken remotely. Difficulties in adhering to protocol defined follow-up are nonetheless inevitable, and will result in an extra ‘chunk’ of missing outcome data occurring for different reasons than would occur without the pandemic. As well as physically missing data, there will be additional concern for participants providing data during Covid-19 when their outcomes are influenced by it. In this situation, a central consideration is the treatment effect to be obtained from the trial (estimand), as this may sometimes require treating some of the collected data as missing. It is therefore recommended that trial statisticians review and update protocol plans for handling missing data in the analysis [2, 3].

Missing data are a critical issue for the analysis of trials, since any statistical method implicitly makes an assumption about the distribution of the unobserved data, and it is not possible to verify this assumption without the missing data. If the assumption is wrong, both the treatment effect estimate and associated estimate of precision will generally be biased. This could have damaging clinical implications. When the proportion of missing data is small, the bias may be negligible, but the larger the proportion, the greater the concern for the scientific credibility of the trial. Nevertheless, the principles underlying the handling of missing data in the analysis remain unchanged: the primary analysis should be conducted under the most plausible assumption as to why the data are missing and their associated distribution, followed by sensitivity analyses under alternative plausible assumptions for the missing data distribution to assess the robustness of conclusions [5–9]. However, the considerations for which missing data assumptions might be most appropriate may differ for trials active during a pandemic. In this article we present a four-step strategy for handling missing outcome data in the analysis of clinical trials whose conduct overlap a pandemic period. The guidance is intended to help statisticians and investigators to maintain the scientific integrity of ongoing trials, despite an unexpected pandemic.

## Methods

We developed a four-step strategy for handling missing outcome data in the analysis of trials whose conduct overlap a pandemic period. The strategy can be used regardless of the outcome type, but our examples will focus on continuous outcomes. This framework was developed to be consistent with the statistical principles outlined in the ICH-E9 guidelines [10] and ICH-E9(R1) addendum on estimands and sensitivity analysis in clinical trials [11]. A particular focus is on carrying out relevant primary and sensitivity analyses to ensure trial results address the question of interest, and are unbiased under plausible assumptions about the missing data. Using Rubin’s classification of missing mechanisms [12], we provide structured guidance to help assist decisions about handling missing data under plausible assumptions. Following an outline of the main issues raised by a pandemic we describe each point of the guidance in turn, which we illustrate using the ASCOT trial, an ophthalmic trial ongoing during Covid-19. We summarise the guidance using a decision tree.

### The ASCOT trial

As an example, we consider the Adjunctive Steroid Combination in Ocular Trauma (ASCOT) trial, a pragmatic, double-blind, multi-centre randomised controlled trial testing whether adjunctive steroid (triamcinolone acetonide), given at the time of surgery for open globe trauma can improve the outcome of surgery [13]. Adults undergoing vitrectomy for open globe trauma were randomised to receive adjunctive intraocular and periocular steroid or standard treatment (no steroid) during surgery in a 1:1 ratio. The primary outcome is improvement in visual acuity, measured using the ETDRS (number of letters read at 4 m and 1 m) in clinic at 6 months. An interim ETDRS measure is recorded at 3 months. ASCOT had completed recruitment and treatment for all participants, but had approximately 10% of participants in follow-up when it was interrupted by the Covid-19 pandemic.

## Results

### A four-step strategy for handling missing outcome data in the context of a pandemic

**Fig. 1:**
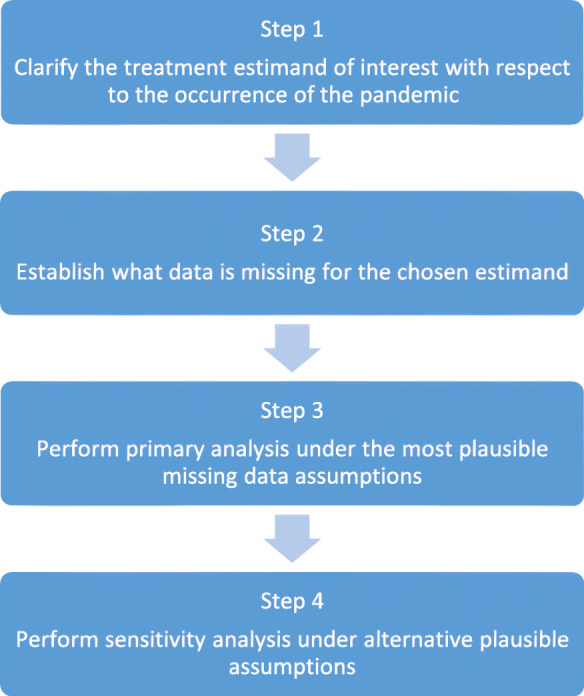
Strategy for handling missing outcome data in clinical trials during a pandemic

Our proposed strategy consists of four key steps, summarised in Fig. 1. We now discuss each point in turn.

### Step 1: clarify the treatment estimand of interest with respect to the occurrence of the pandemic

In any trial, to help ensure the trial answers the question of interest it is important to precisely define the treatment *estimand*. The term *estimand* describes the conceptual quantity that we wish to estimate numerically from a trial. The ICH E9(R1) addendum [11] describes five key attributes that form the description of an estimand. These are: (A) The population; the patients targeted by the scientific question, (B) The treatment condition of interest and the alternative treatment condition(s) e.g. control or placebo, (C) The variables (or endpoint) to be obtained for each patient required to address the scientific question, (D) The specification of how to account for intercurrent events to reflect the scientific question of interest, (E) The population level summary for the variable which provides a basis for a comparison between treatment conditions. For trials ongoing during a pandemic, in line with ICH-E9 [11], we assume a defined treatment estimand prior to the occurrence of the pandemic. Clarification on whether the pandemic impacts the trial estimand is first required. This will inform the missing data problem (step 2) and subsequent handling of missing data (steps 3 and 4).

A pandemic will not typically change estimand attributes A, B, C and E, although there will be exceptions [14, 15]. It is most likely to introduce a number of new intercurrent (post-randomisation) events, which are defined as events affecting the interpretation or existence of trial outcomes [6, 11, 14]. A pandemic may directly affect participant outcomes if they become infected with the underlying disease (e.g. infection with Covid-19). Conversely, a pandemic may indirectly affect participant outcomes due to treatment and standard care disruptions or participant behaviour changes (e.g. due to increased health care demands or participant health care anxieties).

We consider two estimands that may be of interest. The first is the treatment effect in a hypothetical ‘pandemic-free world’, where interest lies in the treatment effect we would have seen had the pandemic not happened. For this estimand, the impacts of the pandemic on the trial (e.g. infection of trial participants, treatment interruptions, etc), whether direct or indirect, obscure our interpretation of the relevant outcome data. As such, this estimand uses a hypothetical strategy to deal with these intercurrent events, where interest lies in the treatment effect that would be seen had these events not occurred.

The second estimand is the treatment effect in a ‘world including a pandemic’, where interest lies in the treatment effect that occurs in the presence of the pandemic. Here, the effects of the pandemic (e.g. infection of trial participants, treatment interruptions, etc) are directly relevant to the estimand. As such, this estimand uses a treatment policy approach to deal with these intercurrent events. We note that this estimand depends both on the degree to which the trial overlapped with the pandemic, and the severity of the pandemic during the overlap period.

For instance, if the same trial were to have started on a different date (and therefore, have a different amount of overlap with the pandemic), the value of the ‘world including a pandemic’ estimand may be quite different for the trial. As such, the generalisability of this estimand should be carefully considered.

The most appropriate estimand of interest will be trial specific and should be guided by the question of key clinical interest [14, 15]. For many trials there may be more than one estimand of interest. As outlined in ICH-E9(R1), following primary analysis, alternative estimands targeting different clinical questions of interest may be explored in supplementary analyses [11]. The following guidance in this four-step strategy can be similarly applied to such supplementary analyses.

After clarifying which estimand is of interest for the analysis at hand (‘world including a pandemic’ vs. ‘pandemic- free world’ estimand), the challenges of a pandemic then surround handing the additional unplanned intercurrent events in a way that remains consistent with the estimand. Some of these pandemic-related intercurrent events may mean that some recorded participant outcomes are no longer relevant for the chosen estimand.

### Step 2: establish what data are missing for the chosen estimand

We can only begin to think about missing data in any trial setting once the treatment estimand has been defined: only once we know exactly what we are aiming to estimate can we know whether we have the data required to estimate it. We define missing data as data that are required to estimate the estimand of interest, but that are unavailable. Some data maybe be physically missing (i.e. not collected) or some observed data may be best treated as missing in the analysis (i.e. set missing by the analyst) if not relevant for the estimand of interest.

For the ‘pandemic-free world’ estimand only participant data that was unaffected by pandemic-related intercurrent events is required for the analysis (i.e. data collected before or after the pandemic, and data collected during the pandemic which was not impacted by pandemic-related infection, treatment interruptions, etc). For such an estimand, if any data are collected from participants who experience pandemic-related intercurrent events (directly or indirectly affecting their outcomes), the affected data are not needed in the analysis and so may be best set missing; including affected participant data will adversely impact the desired estimate. Clinical input will be required to establish how to identify pandemic-related intercurrent events. It has been discussed elsewhere how a pragmatic definition of the pandemic start and end dates (e.g. based on national or local Covid-19 case numbers) may be used to understand the pandemic effect on participant outcomes [15]. Additional data will most likely need to be collected to determine whether participants experienced any pandemic related intercurrent events, for example such as Covid-19 infection, reason for treatment interruption (pandemic related or not). EMA guidelines [3] highlight the importance of capturing deviations resulting from the Covid-19 pandemic, and related reasons, to help distinguish between data affected and unaffected by the pandemic. Pragmatic, uniform, trial specific decisions may be required. We recommend trialists are transparent in reporting all criteria used to inform the analysis. Here, any data unobserved or set missing due to the pandemic creates a missing data problem in the analysis. There may also be missing data from participants whose outcomes were planned to be collected during the pandemic, were not directly or indirectly clinically impacted by the pandemic, but were unobserved due to changes in follow-up procedures or participant engagement. Alongside this, there is the ‘usual’ missing data from participant’s pre- and/or post-pandemic. All such missing data must be handled in line with the targeted ‘pandemic-free world’ estimand.

For the ‘world including a pandemic’ estimand, all participant data pre-, during- and post-pandemic will be required for analysis. However, any participant data not collected still creates a missing data problem that must be addressed in line with the targeted ‘world including a pandemic’ estimand.

We note that if data that are affected by the pandemic, (i.e data from participants who experience a pandemic-related intercurrent event through infection, or treatment interruption, etc) are included in an analysis, then the estimated treatment effect will correspond to a ‘world including a pandemic’ estimand. If this is not desired, and the ‘pandemic-free world’ estimand is required, observed data that are clinically affected by the pandemic in any way (directly or indirectly) should be set to missing. In settings where the pandemic doesn’t clinically affect any participant outcomes (directly or indirectly) the two estimands will coincide and the distinction is not relevant.

### Step 3: perform primary analysis under the most plausible missing data assumptions

Once data that are missing have been established, the most appropriate missing data assumptions can be defined for the primary analysis. The analysis can then be performed using a statistical method that provides unbiased estimation under the chosen assumption. We now review the three general classes of missing data assumptions before providing suggestions on aspects to consider when forming assumptions for missing data in the context of trials impacted by a pandemic.

Missing data assumptions (or mechanisms) can be categorised into three broad types [12]: Missing-completely-at-random (MCAR), Missing-at-random (MAR) and Missing-not-at-random (MNAR). Participant data are MCAR when the reason for the missingness is completely unrelated to the participants’ unobserved outcome or observed values of other recorded variables; the distribution of the missing participant data will look no different to that of the observed participant data. Data are MAR when missingness does not depend on participants unobserved data values once we have taken into account the information in their observed data in the analysis, such as baseline characteristics or, in longitudinal settings, earlier responses. Only once the observed data is conditioned on, will the missing data distribution then look no different to the observed data distribution. In trials, typically MAR will be more likely than MCAR; particularly in longitudinal studies, where conditioning on the partially observed longitudinal outcome data will make the MAR assumption much more plausible. Finally, data are MNAR when the missingness remains dependent on the unobserved values of the data in some form (or on observed data not included in the analysis); the missing participant data will have a potentially substantively different distribution to the observed data. Table 1 summarises the principal methods of analysis for unbiased estimation of a treatment effect (the estimand) under MCAR, MAR and MNAR in clinical trials (not an exhaustive list of options, see [7] or [8] for more detailed guidance).

**Table 1:** Methods for unbiased analysis under MCAR/MAR/MNAR in clinical trials (not an exhaustive listing of options)

Assumption	Method for unbiased estimation	Comments
MCAR	Any complete case analysis	Easy to implement but may not use all the available information in the data. Excludes participants with any missing data.
MAR	Complete case analysis incorporating all variables associated with *both* outcome and missingness	Easy to implement but may not use all the available information in the data. Excludes participants with any missing data. Generally cannot incorporate post-randomisation data.
	Mixed model incorporating all variables associated with *both* outcome and missingness, which may include post-randomisation variables (e.g. for a longitudinal trial a mixed model for repeated measured MMRM)	Earlier response data can be incorporated in the analysis to strengthen/justify MAR. Includes all observed data on each participant. Additional post-randomisation data predictive of both missingness and outcome that are required to be included to justify MAR, but that the treatment estimate (estimand) should not be conditioned on can also be included, however careful model specification is required to do so (e.g. any post-randomisation variables must be included as additional responses in the model with separate means for each treatment group, for detailed guidance see [7]).
	Multiple Imputation incorporating all variables associated with *both* outcome and missingness, which may include post-randomisation variables	Closely approximates a complete case/mixed model analysis when the variables included in the imputation and analysis model match those in the complete case/mixed model analysis. The imputation model must include as a minimum all variables included with the analysis model [16]. Provides a convenient analysis method when conditioning on particular variables is required to justify/strengthen a MAR assumption, but conditioning on these in the analysis is not required/appropriate. This is because variables that are predictive of both outcome and missingness, but that the treatment effect (estimand) should not be adjusted by, can be included in the imputation model and not in the subsequent analysis model.
MNAR	Selection models: Consists of a model for the outcome data and a model for the occurrence of missing data	For example, may consist of a logistic model to model the log odds of response and how this depends on the unobserved outcome. Expert/clinical knowledge is required to inform how the log odds of response depends on the unobserved outcome. Can be fitted using maximum likelihood, or within a Bayesian framework [8].
	Pattern mixture models: Consists of a model for the outcome data for each missing data pattern	Expert/clinical knowledge is required to inform how the outcome data distribution varies for each missing data pattern. Can be fitted using maximum likelihood, within a Bayesian framework or, implemented in a multiple imputation framework, when it is referred to as ‘Controlled Multiple Imputation’ [8, 15]:
	Controlled Multiple Imputation: Combines pattern mixture modelling and multiple imputation (e.g. delta-based or reference-based Multiple Imputation)	Data is imputed multiple times from a pattern mixture model. The analyst has direct ‘control’ over the imputation distribution. For example, in delta-based imputation a numerical offset term, delta, is typically added to the expected value of the missing data to assess the impact of unobserved participants having a worse or better response than of those observed. Reference-based imputation draws imputed values with some reference to the observed data in other groups of the trial, typically in other treatment arms. Different MNAR assumptions can be made for different groups of individuals in the same trial analysis by postulating different distributions for imputation or MAR and MNAR assumptions may be made for different groups [17].

For any trial analysis, care should be taken to ensure an appropriate method is chosen than handles all missing data appropriately for the treatment estimand of interest

The most appropriate assumptions for missing data in the primary analysis will be trial and estimand specific. For all trials where follow-up continues in person, and resources allow, we recommend trialists attempt to contact participants who do not provide follow-up to determine why they are missing data e.g. to identify whether they were directly or indirectly impacted by the pandemic and how this has affected their outcome. This will help inform the most appropriate missing data assumptions. For trials ongoing during a pandemic different missing data assumptions may be best for different groups of participants to ensure assumptions align with the underlying estimand of interest, and we explore this further below.

While there are no assumptions that can be universally recommended, we now provide some structured guidance to help assist the decision on plausible missing data assumptions, if the trial includes:

(i) Participants directly clinically affected by a pandemic (e.g. Covid-19 infection),
(ii) Participants indirectly clinically affected by a pandemic (e.g. changes to treatment/standard care),
(iii) Participants lost to follow-up during pandemic times (outcomes unaffected by pandemic),
(iv) Participants lost to follow-up during non-pandemic times (e.g. pre or post-pandemic).

**Fig. 2:**
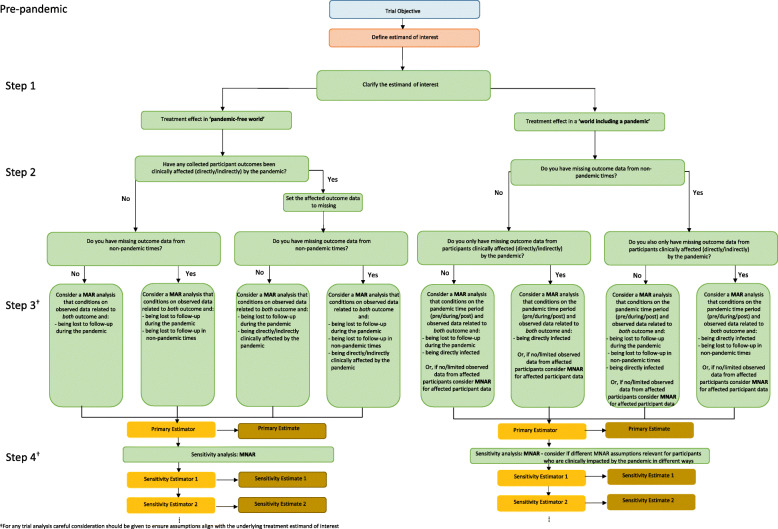
Decision tree to support the appropriate handling of missing outcome data in clinical trials during a pandemic

We address these first for the ‘pandemic-free world’ estimand, followed by the ‘world including a pandemic’ estimand, considering the ASCOT trial as an example. A decision tree summarising our guidance, placed within the ICH E9(R1) framework for aligning trial objectives with conduct and analysis, [10], is provided in Fig. 2 for trialists to follow to support the selection of appropriate missing data assumptions.

### ‘Pandemic- free world’ estimand

#### Participants directly and indirectly clinically affected by a pandemic (i) and (ii)

Outcome data that are clinically affected by a pandemic either directly (e.g. via participant infection with Covid-19), or indirectly, are treated as missing for the ‘pandemic-free world’ estimand and can be handled in the same way as each other. For such data, an MAR assumption —conditional on randomised treatment arm and all observed variables expected to be associated with *both* the trial outcome and being missing (i.e. being directly or indirectly affected) — may be the most reasonable assumption for the primary analysis. Predictors of both missingness and outcome could include baseline participant characteristics (e.g. in ASCOT baseline vision, sex, comorbidities) and/or earlier observed data under pre-pandemic times (e.g. 3 month vision) or during−/post-pandemic times provided these are not affected by pandemic-related intercurrent events. This will assume the missing outcomes for those affected would have been similar to those of participants observed pre- pandemic (and if relevant those observed during or post the pandemic era, who did not experience any pandemic-related intercurrent events and are therefore not affected by the pandemic in any way) with the same values of the observed data, thus enabling estimation of the treatment effect in a pandemic free world.

If it is not required to estimate the treatment estimand conditionally on one or more of the observed variables that are related to both outcome and missingness, for example if a *comorbidity* was expected to be related to both, analysis options (not exhaustive) include using either (a) a carefully constructed mixed model [7, 8] or (b) multiple imputation where the *comorbidity* can be included in the imputation model and not the analysis model [7, 8, 16].

#### Participants lost to follow-up during pandemic times (iii)

Follow-up may continue for participants whose trials outcomes are not impacted directly or indirectly during a pandemic (remotely or in person), but only some of their outcomes may be observed. Here, relative to non-pandemic times, there may be different factors that are expected to be associated with both outcome being missing (due to pandemic follow-up interruptions) and the trial outcome to consider to justify an MAR assumption. These factors may differ depending on the precise mode of follow-up during the pandemic.

For example, for ASCOT participants not infected by the pandemic (assuming the pandemic has no other indirect impacts on participants 6 month vision) if in-person follow-up continues, age may become an important factor to incorporate in the MAR assumption as strongly related to outcome: while prior to the pandemic, age was not associated with both missingness and outcome (only with the outcome, 6 month vision), due to the pandemic older participants may become more likely to stay at home to avoid increased risks of infection. The dataset may consequently end up having a higher rate of missing older people (who are unaffected by the pandemic, but have poorer vision simply because of age) compared to younger people (who are unaffected by the pandemic and may have better vision simply because of age).

In some special cases there may be no observed participant data during the pandemic because follow-up stops for everyone. This might occur when all trial participant outcomes would be impacted (directly or indirectly) by the pandemic. Or, follow-up may stop for all — regardless of any impacts on trial outcomes — for practical/logistical reasons (e.g. clinical staff redeployed). As the halting of follow-up impacts all participants equally, there will likely be no pandemic-specific factors associated with both missingness and outcome (relative to pre-pandemic times) to incorporate for participants lost to follow-up during the pandemic for the ‘pandemic-free world’ estimand. Here MAR conditional on predictors of outcome and missingness expected in non-pandemic times would likely be the most appropriate assumption. Where follow-up stops for all participants, unobserved data during this period could alternatively be considered MCAR, but this would only be reasonable in the absence of any time trends within the participant data. In some trial settings there may be underlying time trends, for example, due to seasonal diseases (winter deaths, infectious diseases etc) or changing severity of disease recruited (as trial teams become more confident) that will create a MAR mechanism. Since the absence of such time trends is a fairly strong assumption, where follow-up stops for all participants, we generally recommend performing analysis under a MAR assumption, conditional on predictors of outcome and missingness expected in non-pandemic time.

#### Participants lost to follow-up during non-pandemic times (iv)

In ASCOT, and likely in any trial overlapping the pandemic, there will inevitably also be data missing from participants in non-pandemic times. Trialists should consider whether the same missing data assumption is relevant for data missing pre- (or post-) pandemic and during the pandemic.

Where prior to the pandemic, a reduced set of factors related to outcome and missingness were considered appropriate for analysis under MAR (e.g., in ASCOT, treatment arm, baseline vision and 3 month vision), analysis under MAR including an enlarged set of factors (to also handle participants of type (i), (ii) and/or (iii), e.g. also including sex, comorbidities and/or age) is suggested. Alternatively, multiple imputation could proceed separately for the two groups (not affected and affected by the pandemic) or for individuals with missing data of type (i), (ii), (iii) and (iv).

In summary, for the ‘pandemic-free world estimand’ a carefully constructed enlarged MAR assumption (including all observed data expected to be related to both missingness and outcome across the trial participants) will often be most appropriate for primary analysis.

### ‘World including a pandemic’ estimand

In a world including a pandemic, participant outcomes that are clinically affected by the pandemic (i. directly with the disease or ii. indirectly via changes to treatment/standard care) may either be observed – or expected – to be quite different to those observable under non-pandemic circumstances. To estimate the ‘world including a pandemic’ estimand, assumptions must be made for any participant data not collected during the pandemic era in a manner consistent to what would have been seen in the pandemic world; similarly, pre−/post-pandemic missing data must also be handled in a manner consistent with pre−/post- pandemic times. We now provide some suggestions on aspects to consider when forming missing data assumptions for this estimand.

#### Participants directly clinically affected by a pandemic (i)

Analysis under MAR, including an indicator of direct pandemic impact e.g. Covid-19 infection status, and all observed data expected to be associated with both trial outcome and missingness (e.g. treatment, risk factors for being impacted by Covid-19 and the vision outcome such as age or diabetes), may be most relevant for the data of participants directly impacted in primary analysis – provided, given the observed factors, the distribution of the unobserved outcome values will be the same as for those observed and directly impacted.

If there are, however, no observed outcome data from directly impacted participants, or if it is not anticipated that Covid-19 infection status will allow adequate modelling of the distribution of the missing outcomes for other Covid-19 infected participants then a MNAR assumption may be more appropriate for the data of participants directly impacted. A strong justification must accompany any MNAR assumption. It is most likely that a direct pandemic impact, i.e. Covid-19 infection, will have a negative impact on the trial outcome of interest, though the reverse is of course possible. As a worst case scenario, one could assume that all infected participants behaved like those observed in the trial’s standard care/control arm, to reflect no treatment benefit (e.g. a jump-to-reference assumption) [18]. Or for the infected participants, one could assume a worse outcome than observed for those in their treatment arm by a particular quantity. For example, in ASCOT, suppose Covid-19 infection negatively impacts vision in a way that is not captured by any observed participant characteristics, we could assume any unobserved infected participant has an outcome 20% worse than for those observed (under MAR) in their treatment arm. A discussion between clinicians, researchers on the ground interacting with the Covid-19 infected participants and statisticians should occur to ensure appropriate, justifiable, MNAR assumptions are chosen.

#### Participants indirectly affected by a pandemic (ii)

Analysis under MAR given the pandemic time period (e.g. during/pre−/post-) and observed variables anticipated to be associated with both the trial outcome and missingness may be most relevant in primary analysis for the data of participants indirectly impacted. If indirect impacts fluctuate over the pandemic period (e.g. the standard of care over a pandemic period changes over time due to fluctuating health care demands) there may be more than just one time point factor to incorporate in the missing data assumptions.

Where a trial data set also includes observed data from participants who are directly impacted by the pandemic, and outcome values of those directly and indirectly affected are impacted in different ways, it may also be relevant to include infection status within the missing data assumption.

If there are no observed outcome data from indirectly impacted participants, or if there are no observed variables that along with the pandemic time point can be used to fully model the outcome distribution for indirectly affected participants with missing data, an MNAR assumption may be more appropriate for the data of participants indirectly impacted by a pandemic. Careful thought would need to be given as to what the missing outcomes might have looked like in the pandemic world and a thorough justification should be provided alongside any employed MNAR assumption.

#### Participants lost to follow-up during pandemic times (iii)

For the data of participants whose outcomes are not directly or indirectly impacted by the pandemic, but who have missing data during the pandemic (e.g. participants in ASCOT who decide not to attend the 6 month follow-up during Covid-19 to avoid travel and risk of infection) analysis under MAR given observed data anticipated to be related to both outcome and missingness may be most relevant; which may differ depending on the precise mode of follow-up during the pandemic.

Where the trial data set also includes observed participant data that are directly and/or indirectly clinically affected by the pandemic it will be essential to also include infection status and/or pandemic time period (pre/during/post) within the missing data assumption for primary analysis for the data of participants impacted by outcome interruptions only (i.e. for those lost to follow-up during the pandemic). This will ensure the missing data for the non-affected participants is modelled based upon the observed non-affected participants (and not the observed clinically affected participants).

#### Participants lost to follow-up during non-pandemic times (iv)

Trialists should consider whether the same missing data assumption is relevant for data missing pre- (or post-) pandemic due to loss to follow-up. Analysis under MAR including an enlarged set of factors (to also handle participants with types (i), (ii) and/or (iii), e.g. also including diabetes, age, infection status, pandemic time point as relevant) may be suitable to handle loss to follow-up outside pandemic times.

Of note, participant characteristics might in some cases be related to both outcome and missingness in the during and non-pandemic time periods, but in different ways. If this is an issue we recommend incorporating interactions between time periods (during/pre/post pandemic) and the affected characteristics in any missing data assumption. Similarly, in some settings participant characteristics might be related to both outcome and missingness but in different ways for those directly infected by the pandemic versus those not. If so trialists should consider incorporating interactions between infection status and the corresponding participant characteristics in the missing data assumption.

In summary, for the ‘world including a pandemic’ estimand a carefully constructed enlarged MAR assumption, involving all observed data expected to be related to both missingness and outcome across the trial participants, potentially including pandemic time period (pre/during/post) and/or participant pandemic infection status where relevant is likely to be the most appropriate starting point for primary analysis. In some cases, an MNAR assumption may be more appropriate for participant data that are directly or indirectly clinically affected by the pandemic, however strong justification of the chosen MNAR assumption would be required. In the next section, we highlight an accessible method for analysis when different missing data assumptions are required for different participants in the same trial analysis.

Of course, a pandemic’s clinical impact (direct or indirect) may be unknown for all or some participants in some trials. Where it is unknown for all participants (e.g. Covid-19 infection status not collected) it will not be possible to condition the missingness on Covid-19 infection status, but the pandemic time period (pre/during/post) can still be incorporated when targeting the ‘world including a pandemic’ estimand.

### Analysis when different missing data assumptions are required for different participants

One method which enables analysis under a variety of missing data assumptions is *Controlled Multiple Imputation* (MI) [17, 19]. Controlled MI means the analyst exerts direct ‘control’ over the imputation distribution. Unobserved data are then imputed under the specified distribution multiple times to obtain multiple complete data sets. These are each analysed with the analysis model of interest that would have been used in the absence of missing data. Results are then combined across imputed data sets (using Rubin’s rules [20]) to provide a single treatment estimate which appropriately takes into account all the uncertainty associated with the unseen values, given the postulated distribution.

We discussed above how different missing data assumptions may sometimes be required for different individuals in the same trial analysis. Controlled multiple imputation provides an accessible tool for this. Different distributions for the missing data of different groups of individuals (e.g. MAR and MNAR) can be postulated for data imputation. For analysis under MNAR, to assess the impact of unobserved participants having a worse or better response than those observed, missing participant data can be imputed assuming the distribution of the observed data (i.e. MAR distribution) +/− a specified numerical location shift. This is known as delta-based multiple imputation. Alternatively missing participant data may be drawn from a distribution that is formed with some reference to observed data in other groups of the trial, typically in other treatment arms. This is known as reference based multiple imputation [18]. For example, in a two-arm placebo-controlled trial, participants with missing data in the active arm can be imputed to follow the distribution of the placebo arm, assuming no treatment benefit following drop-out (referred to as a *jump-to-reference* imputation). Delta and reference based multiple imputation methods can been implemented with continuous [7, 17, 18], binary [21, 22], ordinal [23], count [24–26] and survival data [27–30].

An accessible tutorial provides a more detailed practical overview of controlled MI procedures, with worked examples and Stata code, where different assumptions are incorporated in one analysis [17].

Controlled multiple imputation also provides an accessible, flexible tool for contextually relevant sensitivity analysis which is the next and final key step when handling missing outcome data during a pandemic. We have previously shown that the aforementioned controlled procedures (delta- and reference-based multiple imputation) provide valid inference, as the proportion of information lost due to missing data under MAR is approximately preserved in the analysis [31]; That is *information anchored* inference will be obtained.

### Step 4: perform sensitivity analysis under alternative plausible assumptions

Any missing data assumption is unverifiable, so sensitivity analyses under alternative plausible missing data assumptions should be conducted, regardless of the type of missing data assumption employed for primary analysis. Sensitivity analysis should address the same question as the primary analysis [32]. If it can be shown that the inference is robust to different missing data assumptions then this will provide confidence in the conclusions despite the missing data. If conclusions vary in sensitivity analysis it is equally important to demonstrate under what conditions results look different to ensure misleading conclusions are not drawn. As described above, a MAR assumption will be most plausible for primary analysis in the majority of scenarios. However MAR is still a strong unverifiable assumption. For both the ‘pandemic- free world’ and ‘world including a pandemic’ estimand it will be important to conduct sensitivity analyses under alternative plausible MNAR assumptions, which have MAR as a departure point and align with the chosen estimand.

The challenge is that there will be numerous ways in which the unobserved data could be modelled, so where should one start? One option is to use the MAR distribution as a departure point. The MAR data distribution provides an unambiguous starting point for MNAR exploration. For example, starting with the specification of the conditional data distribution implied by MAR, one can perform sensitivity analysis exploring departures from MAR by shifting the parameter values of the distribution in a contextually relevant manner for the chosen estimand. As introduced above, an analysis under such an assumption can be accessibly conducted using ‘delta-method’ multiple imputation.

For the ‘pandemic free world’ estimand, it cannot be ruled out that —in the absence of a pandemic—participants with missing data would have actually have had worse/or better outcomes than those observed in the trial. This is why conducting sensitivity analysis using a series of shifts in the MAR distribution may be a particularly useful approach to demonstrate the robustness of conclusions here.

For the ‘world including a pandemic’ estimand; depending on the underlying disease, it may be more likely that those affected by the pandemic —or also those that decide not to attend follow-up visits (in person or remotely) — had poorer outcomes than those observed in pandemic times. This MNAR scenario becomes more likely in the context of a pandemic, as a greater number of participants in poorer health may stay at home to avoid additional pandemic implications. Or, depending on the trial context, it may be healthier participants who stay at home — since they feel they don’t have an essential need for clinical follow-up. Starting with an appropriate MAR assumption (conditional on factors in the pandemic time period, pandemic infection status and other predictors of response in a pandemic era) one can assess alternative contextually plausible MNAR assumptions by shifting the parameters of this distribution. The parameter shifts should be relevant to what the unobserved outcomes are hypothesised to look like in the pandemic world.

In any trial setting, careful thought must be given to what are appropriate values for the sensitivity analysis parameters when utilising such an approach. Discussions between clinicians, researchers interacting with the trial participants (those infected and those not infected) and statisticians should take place to help ensure appropriate missing data assumptions are chosen and justified.

The difference between the MAR and MNAR distribution can alternatively be described using within-trial information by reference to other groups in the data in sensitivity analysis. The parameters of the observed data distribution, estimated assuming MAR, can be mixed around, *across* arms rather than within, to form contextually relevant MNAR distributions for the unobserved data. This is referred to as ‘reference based MI.’ Further practical guidance in implementing such sensitivity analysis, including examples and Stata code is provided in [17].

## Discussion

We have provided a four-step strategy to facilitate the handling of missing outcome data in trials ongoing during a pandemic, such as Covid-19. In the first step, the treatment estimand of interest must be clarified (treatment effect in a ‘pandemic-free world’ or the ‘world including a pandemic’). In step 2, the estimand will inform what data are missing. In step 3, primary analysis should then be conducted under the most plausible missing data assumption, or set of assumptions, that align with the trial estimand, followed by sensitivity analysis in step 4, under alternative credible assumptions that also align with the estimand.

Of course, the treatment effect in a ‘pandemic-free world’ or the ‘world including a pandemic’ are not the only potential estimands that may be targeted for trials overlapping a pandemic. For example, an alternative estimand is the treatment effect only ‘during a pandemic’. The most appropriate estimand choice will be trial and context specific. We hope the guidance on assumptions for missing data in this manuscript provide a basis for considerations on other estimands.

Missing data assumptions will be trial and estimand specific and will require careful consideration to ensure assumptions align with the trial estimand clarified in step 1. Multi-disciplinary discussions between statisticians, clinicians, researchers on the ground interacting with participants will be required to determine the appropriate assumptions. As detailed above, analysis under a carefully constructed MAR assumption will in many cases be most plausible for primary analysis, provided all observed data that are expected to be associated with both the outcome and missingness are included. Sensitivity analysis under credible MNAR assumptions is then recommended to demonstrate the robustness or sensitivity of results across scenarios. During a pandemic, MNAR will become more credible in most contexts. Results of sensitivity analyses should be reported alongside the primary result, so that robustness of results can be openly assessed.

To conduct unbiased analysis under MAR in longitudinal trial settings (a) a carefully constructed mixed model [7, 8] or (b) multiple imputation analysis can be used (other options also exists) [7, 8, 16]. The natural flexibility of multiple imputation means it may be a more accessible analysis option where observed data need to be conditioned on to justify the MAR analysis but treatment estimates should be unadjusted for (e.g. interim values of outcome). Using multiple imputation, such observed data can be readily including the imputation model but not the analysis model. Controlled multiple imputation, where the analyst exerts direct control over the imputation distribution (away from MAR), provides an accessible tool for assessing the impact of a variety of contextually relevant MNAR assumptions. Thus multiple imputation may provide a unifying tool for both primary and sensitivity analysis in the pandemic era [17].

Naturally, if a large number of participants experience pandemic-related intercurrent events and their data are either unobserved or set to missing there may be concerns as to whether the ‘pandemic-free world’ estimand can be reliably estimated using *any* missing data method. Trialists will need to carefully consider the available data and the feasibility of obtaining reliable results before conducting analysis with their chosen method of analysis.

The focus of this guidance is on handling missing data. However, when the ‘pandemic-free world’ estimand is of interest and data are affected by the pandemic and observed, setting the affected data to missing and employing an appropriate method to handle the arising missingness is not the only analysis option. Instrumental Variable (IV) methods, used previously to handle treatment switching in trials, provide an alternative analysis option [33, 34]. For example, Rank Preserving Structural Failure Time Models (RPSFTM) for estimating counterfactual survival times or structural nested mean models or more traditional two-stage least squares (2SLS) methods for other data types [35–37]. When a large proportion of the observed data are affected by the pandemic IV methods that appropriately model the observed data may be of value. However, regardless of the analytical approach employed (e.g. missing data approach versus modelling observed data), when a substantial majority of data are impacted by the pandemic any analysis for the pandemic free world estimand will be heavily assumption led. Assumptions will simply be different. Thus, caution will still be required when targeting the ‘pandemic-free world’ and there is a large proportion of affected data be this observed or set missing.

We have focused exclusively on the impact of missing outcome data on treatment effect estimation during Covid-19. In our discussions we did not consider an interaction between Covid-19 initiation and the treatment effect which may be relevant to the ‘world including a pandemic’ estimand*.* EMA guidelines recommend considering treatment effect estimation for the pre-, during and post-Covid-19 periods where relevant [3]. The methods discussed here for handling missing data will be applicable where this interaction is included.

Finally, we note that this four-step strategy could be readily adapted and adopted in non-pandemic settings to handle the occurrence of other unplanned intercurrent events. In step 1 the treatment estimand can be clarified with respect to any unplanned intercurrent event and steps 2, 3, and 4 follow as specified in Fig. 1.

## Conclusions

Missing data issues are likely to be exacerbated in pandemics. We have proposed a four-step strategy to consider and think through the issues raised in a systematic manner. More than ever, careful consideration of missing data assumptions is required. Further, because more data are missing, sensitivity analysis will play a more crucial role than ever in demonstrating the robustness or sensitivity of trial results. Our suggestions will not cover all potential trial settings, but the generic strategy illustrated, by the examples, provides a practical framework for many trials.

## Data Availability

Data sharing is not applicable to this article as no datasets were generated or analysed during the current study.

## Abbreviations

2SLS: Two-stage least squares
ASCOT: Adjunctive steroid combination in ocular trauma trial
Covid-19: Novel coronavirus (SARS-CoV-2) pandemic
EMA: European Medicines Agency
ETDRS: Early treatment diabetic retinopathy study
FDA: Food and Drug Standards Agency
IV: Instrumental variable
MAR: Missing-at-random
MCAR: Missing-completely-at-random
MHRA: The Medicines and Healthcare products regulatory agency
MMRM: Mixed model for repeated measures
MNAR: Missing-not-at-random
MI: Multiple Imputation
RPSFTM: Rank preserving structural failure time models
